# Genetic Selection Based on a Ste6^*^C-HA-Ura3 Substrate Identifies New Cytosolic Quality Control Alleles in *Saccharomyces cerevisiae*

**DOI:** 10.1534/g3.120.401186

**Published:** 2020-04-16

**Authors:** Shu Ning Chan, Rupali Prasad, Paul Matsudaira

**Affiliations:** * Temasek Life Sciences Laboratory, 1 Research Link, National University of Singapore, Singapore 117604,; † National University of Singapore Graduate School for Integrative Sciences and Engineering (NGS), University Hall, Tan Chin Tuan Wing, Level 04, #04-02, 21 Lower Kent Ridge, Singapore 119077, and; ‡ Centre for BioImaging Sciences, Mechanobiology Institute, Department of Biological Sciences, National University of Singapore, 14 Science Drive 4, Singapore 117543

**Keywords:** genetic selection, cytosolic protein quality control, spontaneous mutations

## Abstract

Protein quality control in the cytosol (CytoQC) is an important cellular pathway consisting of a network of components which monitor the folding of cytosolic proteins and ensure the efficient removal of aberrant ones. Our understanding of CytoQC mechanisms is limited in part by the ability of current approaches to identify new genes in the pathway. In this study, we developed a CytoQC reporter substrate, Ste6*C-HA-Ura3, for a new genetic selection of spontaneous CytoQC mutations in the yeast *Saccharomyces cerevisiae*. In addition to UBR1, which encodes for a known CytoQC E3 ligase, we identified six new CytoQC candidates. In the preliminary characterization of two mutants, we found that Doa4 is involved in the degradation of misfolded substrates while Pup2 functions in the selectivity of CytoQC and ERAD substrates. Overall, the strategy demonstrates the potential to identify novel genes and advance our understanding of CytoQC.

Each cellular organelle has a protein quality control (PQC) network that monitors the integrity of protein folding and subunit assembly into complexes by recognizing and removing excess and misfolded proteins and protein aggregates (Hartl *et al.* 2011; Prasad *et al.* 2012; Abrams *et al.* 2014; Balchin *et al.* 2016; Boomsma *et al.* 2016). Collectively, these networks ensure a balance between protein synthesis and degradation and protect the cell from toxicity of aberrant protein structures. Without a properly functioning PQC, the aberrant protein structures accumulate and lead to, in some cases, degenerative diseases such as Alzheimer’s and Parkinson’s (Chiti and Dobson 2006; Knowles *et al.* 2014).

In comparison to the well-studied endoplasmic reticulum (ER) PQC of the budding yeast, *Saccharomyces cerevisiae*, in which misfolded polypeptides are targeted by the ER-associated degradation (ERAD) system (Berner *et al.* 2018), less is known about the mechanisms of PQC in the cytosol (CytoQC). CytoQC monitors cytosolic proteins, including misfolded and mis-localized proteins, and targets them for degradation via the ubiquitin-proteasome system (UPS) (Finley 2011). Misfolded cytosolic substrates are first triaged by a complex of Hsp70 chaperones, cochaperones, and nucleotide exchange factors which maintains protein solubility, prevents aggregation and facilitates refolding (Lee *et al.* 1996; McClellan *et al.* 2005; Park *et al.* 2007; Metzger *et al.* 2008; Kampinga and Craig 2010; Mandal *et al.* 2010; Gowda *et al.* 2013; Summers *et al.* 2013; Abrams *et al.* 2014; Gowda *et al.* 2016). If refolding fails, the misfolded cytosolic proteins are ubiquitinated by E3 ligases of the UPS, such as San1 and Ubr1 (Glickman and Ciechanover 2002; Heck *et al.* 2010; Prasad *et al.* 2010, 2012; Gowda *et al.* 2013; Guerriero *et al.* 2013; Summers *et al.* 2013; Shiber and Ravid 2014; Amm and Wolf 2016). In the final step, the polyubiquitinated proteins are degraded by the 26S proteasome, which is responsible for the turnover of a wider diversity of cellular proteins ranging from folded to misfolded proteins (Voges *et al.* 1999; Goldberg 2003; Prakash *et al.* 2009). However, beyond this overview of the pathway, there are gaps in understanding how the CytoQC mechanism commits proteins to degradation or refolding, mediates the import of substrates into the nucleus, and how the proteasome recognizes cytosolic misfolded substrates. Furthermore, because the degradation delay of different model substrates is partially compensated in mutants of CytoQC (Gowda *et al.* 2013; Summers *et al.* 2013; Prasad *et al.* 2018), there may be additional and possibly redundant components in the pathway.

In contrast to the limited screens of single deletion libraries or from specific searches for functionally-related proteins (Eisele and Wolf 2008; Heck *et al.* 2010; Fang *et al.* 2011; Theodoraki *et al.* 2012; Gowda *et al.* 2013; Fang *et al.* 2014; Comyn *et al.* 2016; Fang *et al.* 2016), we have designed a broad and complementary selection based on a new CytoQC reporter substrate and have identified the E3 ligase Ubr1 and six new CytoQC components. To validate our strategy of the broad selection for uncovering new components of the CytoQC pathway, we have preliminarily characterized two new candidates, the proteasomal alpha subunit Pup2 and deubiquitinase Doa4.

## Materials And Methods

### Yeast strains and media

*Saccharomyces cerevisiae* strains used in this study were in the W303 background (*MAT***a** *ade2**-1*, *his3**-11*, *leu2**-3,112*, *ura3**-1*, *trp1**-1*, *can1**-100*). Yeast transformed with plasmids encoding the different substrates were grown in synthetic complete (SC) media lacking the necessary nutrient(s) for selection. Strains used are listed in Table S1.

### Plasmids generated in the study

Plasmids in this study were constructed following standard cloning protocols (Sambrook *et al.* 1989). Genes were cloned into centromeric vectors expressing selection markers and were sequenced in their entirety using Sanger sequencing for confirmation. The reporter substrate Ste6*C-HA-Ura3 was expressed under a PRC1 promoter in a centromeric plasmid with the ACT1 terminator. CytoQC substrates Ste6*C and ∆ssPrA were expressed under the strong constitutive TDH3 promoter, and ERAD substrates CPY*, Sec61-2 and Ste6* and the wild-type copies of candidate genes were expressed under their respective endogenous promoters. All misfolded substrates have an engineered HA epitope appended to the C-terminus. Lists of the plasmids and oligonucleotide primers used in this study can be found in Tables S2 and S3 respectively.

#### pRP37:

pSW2 was digested with *Bam*HI and *Eco*RI to release the PRC1 promoter. The resulting fragment was ligated into pRP22 (Ste6*C-HA) cut with *Bam*HI and *Eco*RI, generating pRP39. To generate the fusion reporter Ste6*C-HA-Ura3, a 750-bp fragment of Ste6*C-HA was first amplified from pRP22 using primers RP205 and RP52 and digested with *Bam*HI and *Sma*I. Second, a 1.5-kb fragment containing URA3 followed by the *ACT1* terminator sequence was digested with *Sma*I and *Xba*I and released from pDN99. The two fragments were ligated into pRP39 digested with *Bam*HI and *Xba*I to generate pRP37. Plasmids pSW2 and pDN99 were taken from the Davis Ng lab plasmid collection.

#### pSN87:

The PUP2 gene, with its endogenous promoter and terminator, was amplified by PCR using primers SN427 and SN428, digested with *Not*I and *Sal*I, and cloned into the pRS314 vector.

#### pSN59:

The DOA4 gene, with its endogenous promoter and terminator, was amplified by PCR using primers SN295 and SN296, digested with *Not*I and *Xho*I, and cloned into the pRS314 vector.

### Generation of spontaneous mutants

Wild-type (WT) cells expressing the reporter substrate, Ste6*C-HA-Ura3, were streaked for single colonies on non-selective YPD plates. Single parent colonies were picked and each further grown in YPD media overnight and plated on SC-Ura selection plates. Parent colonies streaked on SC-Ura plates which yielded numerous colonies (red plate) were eliminated (Figure S1). Remaining parent colonies grown in YPD media were plated on fresh SC-Ura plates at different dilutions from OD_600_ of 0.1 to 1.0. Single colonies of different widths on the SC-Ura plates were picked across four days from the third day since plating on SC-Ura, up to a week. The rate of spontaneous mutations in CytoQC genes was later calculated to be about 1x10^−6^. The entire selection was performed at 25°.

### Primary selection

Based on the observation of the CytoQC-defective phenotypic growth on SC-Ura plates, colonies from the selection were selected and streaked further on fresh SC-Ura plates for single colonies and on SC-His plates to check for the presence of the reporter plasmid. Isolates that did not retain the reporter plasmid were discarded. The reporter plasmid was also dropped from remaining isolates by growing out on YPD plates and replica plating onto SC-His and SC-Ura plates. Colonies which failed to grow on SC-His and SC-Ura were isolated and retransformed with the reporter plasmid. Strains which failed to display uracil prototrophy after retransformation were discarded. Later, mutants were tested for recessive and dominant alleles by crossing with the WT strain of the opposite mating type. Diploids were tested for uracil prototrophy by growing them on SC-Ura plates. Diploid strains which grew on the SC-Ura plate suggested the allele to be dominant while the opposite confirmed alleles to be recessive. For recessive mutants, tetrad dissection was performed to determine the allelic complexity of the mutants. Only those exhibiting a 2:2 segregation pattern (two grow to two dead on the SC-Ura plate) indicating a single mutant gene were continued. Strains were backcrossed before further analysis.

### Secondary selection for CytoQC defect

Mutants were transformed with centromeric plasmids expressing known CytoQC genes and streaked on SC-Ura plate for prototrophic growth. Only transformed mutants which continued to display prototrophic growth (CytoQC defect not rescued by known genes) were selected for characterization. Selected mutants were assayed based on reporter stabilization using cycloheximide chase assays and Western blot. Finally, the CytoQC defect in selected mutants was confirmed by a delay in degradation of model CytoQC substrate Ste6*C using metabolic pulse chase assays. Confirmed mutants were processed for identification of the CytoQC candidate gene using cloning complementation or whole genome sequencing.

### Genomic DNA extraction and whole genome sequencing

Genomic DNA (gDNA) from yeast was extracted by the phenol-chloroform method following a modified protocol by Huibregtse and Engelke (1991). Extracted gDNA was incubated in 50µg/ml Ribonuclease A (RNaseA) (Sigma Aldrich) for three days at 37° and resuspended in Tris-EDTA buffer before use. The gDNA was sent for whole genome sequencing to the DNA Sequencing Core Facility of the University of Utah and sequenced using Ion Torrent Next-Generation Sequencing machine with a coverage of at least 50X. Basic variant analyses received were further analyzed by comparing the mutants against the WT sample and filtering for mutations with allele frequencies above 90%. Candidate genes were further selected based on uniqueness to individual mutant genomes and the mutation was confirmed with Sanger sequencing using primers flanking the putative mutation site. A wild-type copy of the candidate gene was then cloned into a low-copy centromeric plasmid and transformed into the mutant. A rescue of the CytoQC-defective uracil prototrophic phenotype was observed if the cloned candidate gene was correctly identified to be responsible for the CytoQC defect. File S1 contains the whole genome sequencing results of the identified new mutants.

### Spot dilution assay

Yeast strains were grown to log phase in selection media and diluted to starting dilution of 0.2 OD_600_. Five dilutions of 10X each were prepared starting from 0.2 OD_600_ and 8µl per dilution was spotted on selection plates. Plates were incubated at 30° for growth assays.

### Cycloheximide chase assay

Yeast strains were grown to log phase in selection media at 30°. A total of 8 OD_600_ units of cells per strain was pelleted, resuspended in fresh selection media and incubated at 30° for recovery. For temperature-sensitive mutant strains, strains were grown at 25° and incubated at 37° for recovery. To stop protein synthesis, 200µg/ml of cycloheximide was added and chased for various time points. Cells were collected at each time point and 100% Trichloroacetic acid (TCA) was added to give a final concentration of 10% TCA. Cell lysates were prepared by TCA precipitation and proteins were detected by immunoblot.

### Immunoblot

Yeast were grown to log phase in selection media and total lysate was harvested. Cells were lysed mechanically using bead beating with zirconium beads (BioSpec Products). Total protein extract was prepared using TCA precipitation. A portion of the total protein extract was separated on 4–15% gradient SDS-PAGE gel and transferred onto a PVDF membrane (Biorad #1704156) or nitrocellulose membrane (Biorad #1704159). The membrane was incubated with Odyssey blocking buffer (LI-COR) for one hour at room temperature. For probing with anti-ubiquitin, an Invitrogen 4–20% gradient gel was used, and the membrane was autoclaved prior to blocking. The membrane was incubated with appropriate primary antibodies for one hour at room temperature or overnight at 4°. Primary antibodies used were mouse monoclonal anti-HA11 (BioLegend), mouse monoclonal anti-ubiquitin (Invitrogen Ubi-1 #13-1600), rabbit anti-URA3 serum (Davis Ng Lab), mouse monoclonal anti-PGK1 (Invitrogen) and rabbit anti-Kar2 serum (provided by P. Walter (University of California, San Francisco)).

The membrane was then washed thrice with 1X phosphate buffer solution with 0.1% Tween 20 and incubated with secondary antibodies goat anti-rabbit IRDye680 and anti-mouse IRDye800 (LI-COR) at 1:15000 dilution for one hour at room temperature. After washing, the membrane was scanned using the Odyssey Li-Cor Imaging System and the bands were quantified with Odyssey V3.0 software.

### Metabolic labeling (pulse chase assay) and immunoprecipitation

Pulse chase labeling and immunoprecipitation was performed as described previously (Zhang *et al.* 2017). Briefly, cells were labeled with 27.5µCi/OD_600_ of [^35^S]-methionine/cysteine (EasyTag EXPRESS^35^S Protein Labeling Mix (14mCi) #NEG772014MC) for 5 or 10min as stated. After which, 10µl/OD_600_ of cold chase media (2mM methionine, 2mM cysteine) was added and equal aliquots of cells were collected at specific time points and mixed with 100% TCA to give a final concentration of 10% TCA. Cells were mechanically lysed with two rounds of bead-beating and TCA-insoluble precipitates were centrifuged and resuspended in TCA resuspension buffer. Equal aliquots of this resuspension were mixed with 4ml of biodegradable scintillation counting cocktail (Bio-Safe NA, Research Products International Corp.) for TCA precipitable counts using a liquid scintillation analyzer (Perkin Elmer Tri-Carb 4810 TR). The resulting radioactivity counts were used for normalization with the first time point for each sample.

Normalized volumes of proteins were immunoprecipitated with the monoclonal anti-HA11 antibody (BioLegend) and Protein A-Sepharose beads (Sigma Aldrich). Proteins were resolved on SDS-PAGE, and the gel dried and exposed to a phosphoscreen for 18-72 hr. The screen was scanned using the Storage Phosphor mode of the Typhoon Variable Mode Imager 9200 or Amersham Typhoon IP Biomolecular Imager and quantified with the ImageQuant TL Software. The data points on the graphs are reflective of at least three independent experiments with error bars indicating the standard deviation of the mean.

### Substrate ubiquitination assay

A total of 10 OD_600_ units of yeast grown to log phase in selection media was harvested and lysed mechanically using zirconium beads (BioSpec Products). Cell lysates were precipitated and prepared with TCA precipitation as above. Small equal volumes of each lysate sample were resolved on a 4–15% gradient SDS-PAGE gel (Biorad) and transferred onto a nitrocellulose membrane (Biorad) for quantification and normalization of HA-tagged substrates in each sample with immunoblot. Normalized volumes of proteins were immunoprecipitated with the anti-HA affinity matrix (Roche). Proteins were resolved on a 4–15% gradient SDS-PAGE gel (Biorad) and transferred onto a nitrocellulose membrane (Biorad), autoclaved and immunoblotted for polyubiquitinated substrates. The membrane was scanned with the Odyssey Li-Cor Imaging System and the bands were quantified with ImageJ (Schindelin *et al.* 2012).

### Indirect immunofluorescence

Indirect immunofluorescence was performed as described previously (Prasad *et al.* 2018) with minor modifications. Briefly, yeast strains were grown to log phase and fixed with 3.7% formaldehyde (final concentration) at 30° for 90min. Cells were washed with 0.1M potassium phosphate (K_3_PO_4_) and spheroplasted with zymolyase digestion (1mg/ml zymolyase 20T (US Biological), 0.1M K_3_PO_4_, 1.2M sorbitol) at room temperature for 30min. Spheroplasts were applied to alternate wells of a poly-_L_-lysine-coated slide. Slides were immersed in ice-cold PBS for 3min, followed by ice-cold methanol for 6min and ice-cold acetone for 30s. Cells were incubated with blocking buffer (5% BSA in PBS with 0.05% Tween20) at room temperature for 30min, followed by primary antibody incubation overnight at 4°. Primary antibodies used were mouse anti-HA11 (BioLegend) and polyclonal rabbit anti-Kar2 serum (provided by P. Walter (University of California, San Francisco)) at 1:200 and 1:1000 dilutions respectively. Slides were washed twice with PBS and incubated with secondary antibodies in the dark at room temperature for 90min. Secondary antibodies used were anti-mouse Alexa488 and anti-rabbit Alexa546 (Molecular Probes) at 1:500 dilution. Nuclei were visualized using 1mg/ml DAPI (4’,6-diamidino-2-phenylindole, Sigma Aldrich D9542) and samples were examined at room temperature with the FLUOVIEW FV3000 Olympus inverted confocal microscope with a 100x/1.4NA Olympus U Plans Apo objective oil lens. Images were archived and processed with the FV31S-SW/DT software (Olympus) and Adobe Photoshop.

### Molecular dynamics simulation

The protein structure of WT Pup2 was obtained from the yeast 20S proteasome structure (PDB entry 1RYP, Groll *et al.* 1997). The point mutation at residue 101 (Leu101Pro) on Pup2 was introduced into the 1RYP structure (The PyMOL Molecular Graphics System, Version 2.0 Schrödinger, LLC.) and simulated by molecular dynamics the yeast proteasome structure containing the *pup2**-10* mutated subunit. Both WT Pup2 and *pup2**-10* proteasome structures were visualized with the Visual Molecular Dynamics (VMD) software (Humphrey *et al.* 1996), and annotated with Adobe Illustrator.

### Data availability

*S. cerevisiae* strains listed in Table S1 and the plasmids listed in Table S2 are available upon request. The whole genome sequencing data for the identified mutants are found in File S1. All supplementary materials (four tables and seven figures) have been uploaded to figshare: https://figshare.com/s/bc76cfa8b36b981c8d02.

## Results

### Ste6^*^C-HA-Ura3 is a bona fide CytoQC substrate

**Figure 1:**
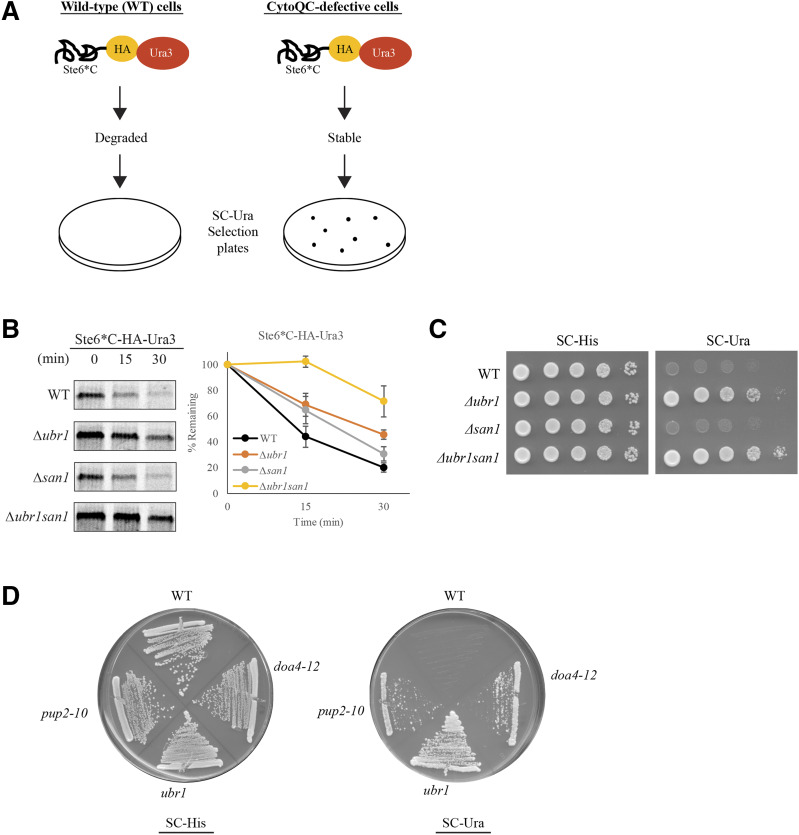
Ste6*C-HA-Ura3 is a *bona fide* CytoQC substrate. (A) Ste6*C-HA-Ura3 serves as a reporter substrate based on the observation of a growth phenotype on SC-Ura selection plates in CytoQC-defective strains. It consists of a misfolded portion (Ste6*C), a HA-tag, and Ura3 as the reporter. (B) Degradation delay of Ste6*C-HA-Ura3 observed in *∆**ubr1* and exacerbated in *∆**ubr1**san1*. Strains were pulsed with 35S-Met/Cys for 10min followed by chase for the indicated time points. (C) CytoQC-defective phenotype was observed for *∆**ubr1* and *∆**ubr1**san1* by growth on SC-Ura plates. Equal numbers of WT and deletion strains expressing the reporter substrate were spotted as 10-fold serial dilutions on SC-His and SC-Ura plates. SC-His plate selects for the reporter plasmid and SC-Ura plate selects for the CytoQC-defective phenotype. (D) Representative figure of the phenotypic growth displayed by WT and CytoQC-defective mutants expressing Ste6*C-HA-Ura3 on SC-His and SC-Ura plates.

To uncover new candidates of CytoQC, we designed the reporter substrate, Ste6*C-HA-Ura3, a fusion protein combining a misfolded substrate and a folded reporter. The misfolded domain expresses the model CytoQC substrate Ste6*C, which requires the E3 ligases Ubr1 and San1 for proteasomal degradation (Prasad *et al.* 2012). Ste6*C is the C-terminus and cytosol-localized form of ERAD substrate Ste6-166 (Ste6*), an ABC transporter with a point mutation that causes it to misfold (Loayza *et al.* 1998). The folded domain expresses the reporter protein from a functional URA3 gene encoding for orotidine-5′-phosphate (OMP) decarboxylase, an enzyme involved in uracil biosynthesis. By combining the two domains, Ste6*C-HA-Ura3 is designed to identify CytoQC-defective mutants based on the prototrophic growth of mutants on SC-Ura agar plates (Figure 1A).

We first validated Ste6*C-HA-Ura3 as a CytoQC substrate by expressing it in deletion strains of known components, Ubr1 and San1. Consistent with the model substrate Ste6*C (Prasad *et al.* 2012), the single deletion *∆**ubr1* strain expressing Ste6*C-HA-Ura3 displayed a degradation delay accompanied by growth on SC-Ura plates (Figure 1B and C). This confirms that Ste6*C-HA-Ura3 is also degraded by the Ubr1-dependent pathway of CytoQC. In addition to *∆**ubr1*, Ste6*C-HA-Ura3 was almost completely stabilized in the double deletion *∆**ubr1**san1* strain (Figure 1B). Likewise, *∆**ubr1**san1* expressing Ste6*C-HA-Ura3 showed faster prototrophic growth on SC-Ura (Figure 1C), suggesting that San1 also plays a role in the degradation of the reporter substrate.

Indeed, when Ste6*C-HA-Ura3 was expressed in the WT strain, the functional CytoQC mechanism recognizes the misfolded domain Ste6*C and targets the whole reporter substrate for degradation, and thus, the strain failed to grow on SC-Ura plates (Figure 1D). Conversely, in CytoQC-defective mutants (*doa4**-12*, *ubr1*, *pup2**-10*), Ste6*C-HA-Ura3 is sufficiently stabilized, allowing the Ura3 reporter protein product to give an uracil prototrophic growth phenotype (Ura+) on SC-Ura plates (Figure 1D). Together, these data provide evidence that Ste6*C-HA-Ura3 is a *bona fide* substrate of CytoQC.

### Genetic selection based on spontaneous mutations identifies new components of CytoQC

With Ste6*C-HA-Ura3, a genome-wide CytoQC mutant selection, based on mutations which arise spontaneously during replication, was set up to identify new components of the CytoQC pathway (Figure S1). We isolated single colonies displaying the CytoQC-defective phenotype on SC-Ura plates and performed primary and secondary selections to eliminate false positive and previously characterized CytoQC mutants. These single colonies were first screened by retransforming the reporter plasmid and checking for the Ura+ phenotype to eliminate mutants with revert mutations of *ura3**-1* or *cis* mutations in the plasmid. As the main goal of the spontaneous mutation selection was to identify new CytoQC components, we next eliminated mutants which harbored mutation(s) in known CytoQC genes. In this subsequent selection, the true mutants were sequentially transformed with low-copy plasmids each expressing a known CytoQC gene and checked for a rescue of the Ura+ phenotype. Transformed mutants which continued to display the Ura+ phenotype were further selected. Finally, these mutants with alleles of interest were finalized based on the stabilization of Ste6*C-HA-Ura3 by cycloheximide chase and immunoblot analyses (Figure S2), and the degradation kinetics of established CytoQC substrates by pulse chase assays. These alleles were identified by whole genome sequencing. A tabulation of the number of isolates at each step of selection can be found in Table S4.

In total, we have identified spontaneous mutant alleles of UBR1 and six new CytoQC genes which encode for Doa4, a deubiquitinase, and proteasomal subunits which span both the regulatory 19S and core 20S proteasomes such as Rpt3 and Pup2 respectively (Table 1 and File S1). Based on the degradation kinetics of model misfolded substrates in the mutants (Figure S3), PUP2 and DOA4 were chosen for validation and characterization.

**Table 1:** Identified mutant alleles of the new CytoQC genes. Putative function of the genes is obtained from the Saccharomyces Genome Database

Candidate Gene	Putative Function	Mutation site	Temperature sensitivity (TS)
RPN11	19S proteasomal subunit, metalloprotease involved in deubiquitination	356C > T (Ser119Phe)	TS
RPT3	19S proteasomal subunit, AAA ATPase	626T > A (Val209Asp)	TS
RPT5	19S proteasomal subunit, AAA ATPase	846A > T (Glu282Asp)	Non-TS
PUP2	20S proteasomal core alpha-5 subunit	302T > C (Leu101Pro)	Non-TS
PRE7	20S proteasomal core beta-6 subunit	71A > C (Tyr24Ser)	TS
DOA4	Ubiquitin hydrolase	1876C > T (Gln626Stop)	Non-TS
		986C > A (Ser329Stop)	Non-TS

### Pup2 primarily affects the degradation of misfolded CytoQC substrates

**Figure 2:**
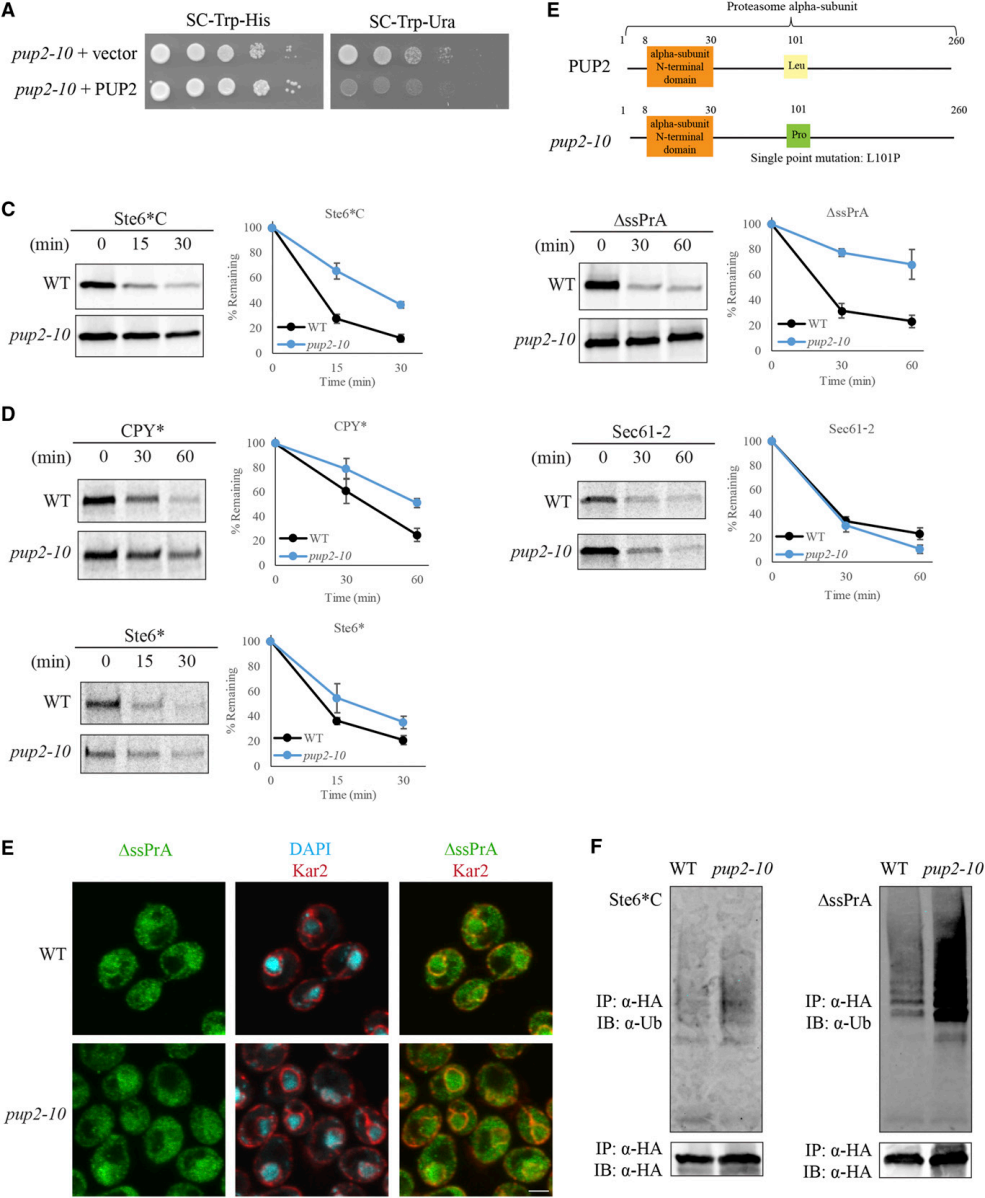
Pup2 predominantly affects CytoQC compared to ERAD. (A) Rescue of the CytoQC-defective phenotype observed with the exogenous expression of wild-type Pup2 (PUP2) in the *pup2**-10* mutant. Equal numbers of each strain were spotted as described in Figure 1C with SC-Trp-His and SC-Trp-Ura plates for selection. Vector: empty vector. (B) Missense mutation in spontaneous mutant *pup2**-10* is present at residue 101, replacing Leucine for Proline. (C-D) Degradation kinetics of CytoQC and ERAD substrates were determined by pulse chase analyses. Strains were pulsed with 35S-Met/Cys for 5min for Ste6*C and Ste6* and 10min for ∆ssPrA, CPY* and Sec61-2, followed by chase for the indicated time points. (E) CytoQC substrate ∆ssPrA is localized predominantly in the nucleus in WT and *pup2**-10*. Substrates were detected with anti-HA antibody (green). The ER and nuclear envelope were visualized with anti-Kar2 antiserum (red). Nucleus was visualized with DAPI staining. Scale bar: 2µm. (F) Accumulation of polyubiquitinated Ste6*C and ΔssPrA was observed in *pup2**-10* mutant compared to WT. Misfolded cytosolic substrates expressed in WT and *pup2**-10* were immunoprecipitated (IP) by anti-HA antibody, resolved by SDS-PAGE and analyzed by immunoblotting (IB) with anti-ubiquitin antibody to detect polyubiquitinated substrates.

PUP2 is an essential gene which encodes for the alpha-5 subunit of the 20S core proteasome (Georgatsou *et al.* 1992; Chen and Hochstrasser 1995). The mutant obtained, *pup2**-10*, is not temperature sensitive and harbors a missense mutation at residue 101 (Figure 2A, 2B and S4). To characterize this mutant, we investigated the degradation kinetics of model CytoQC misfolded substrates, Ste6*C and ∆ssPrA (Prasad *et al.* 2010, 2012), and found them significantly delayed in *pup2**-10*, comparable to the other spontaneous mutants (Figure 2C and S3). Because *pup2**-10* is a proteasome mutant, we assayed the degradation of ERAD substrates, CPY*, Sec61-2, and Ste6* (Vashist and Ng 2004), to determine whether other PQC pathways are affected. Although the *pup2**-10* mutant was expected to display slower degradation than WT, the degradation of CPY* in *pup2**-10* was less delayed than in other spontaneous mutants (Figure 2D and 4D). Degradation of Sec61-2 and Ste6* was also less delayed in *pup2**-10* (Figure 2D and 4D). This smaller difference in degradation for ERAD substrates suggested that Pup2 is only partially required for ERAD and predominantly affects CytoQC substrates.

To determine what step in the CytoQC pathway is affected by *pup2**-10*, we tested whether substrate trafficking of ∆ssPrA into the nucleus is impaired. ∆ssPrA is a model CytoQC substrate that has been shown to import into the nucleus for degradation (Prasad *et al.* 2010, 2018). In our immunofluorescence micrographs, ∆ssPrA was localized predominantly in the nucleus in both WT and the *pup2**-10* mutant (Figure 2E), indicating that nuclear import is not hindered and therefore does not contribute to the degradation delay of substrates.

Once imported, misfolded CytoQC substrates are recognized by the major E3 ligases, San1 and Ubr1, for polyubiquitination, followed by proteasomal degradation (Prasad *et al.* 2010, 2012, 2018). We thus examined the efficiency of this step by checking the ubiquitination status of CytoQC substrates. As expected for proteasomal mutants, a slight accumulation of polyubiquitinated species of Ste6*C and ∆ssPrA was observed in *pup2**-10* compared to WT (Figure 2F). Similarly, an accumulation of polyubiquitinated species of ERAD substrate Sec61-2 was also observed in *pup2**-10* (Figure S5). This accumulation in the mutant suggests that polyubiquitination of substrates in *pup2**-10* is not hindered and that the defect in the QC pathway is likely downstream. Therefore, although Pup2 is a core proteasome subunit, the results interestingly suggest that degradative function of the proteasome is affected in CytoQC but likely less so in ERAD.

### MD simulation predicts the pup2-10 mutation affects the 20S proteasome structure

**Figure 3:**
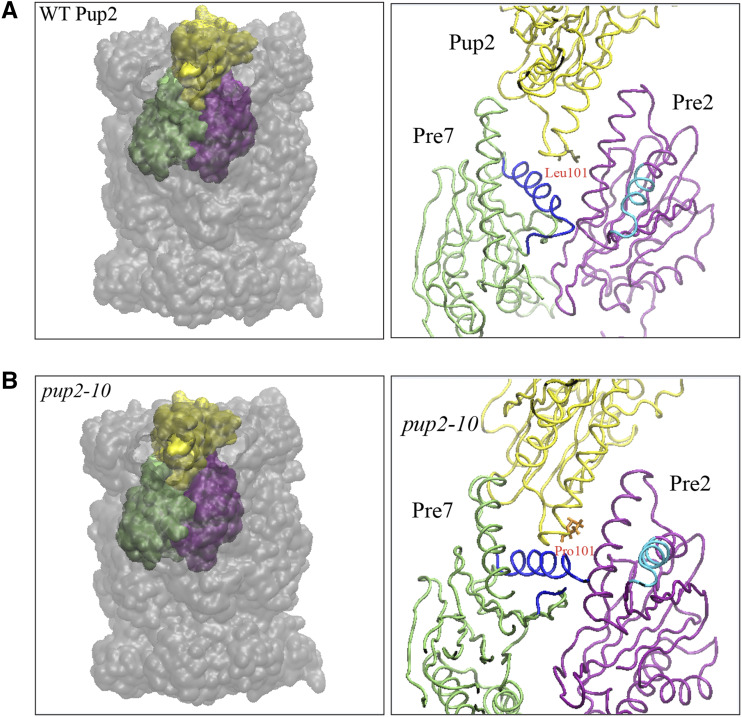
Molecular dynamics simulation of the missense mutation in *pup2**-10* predicts changes in orientation of the Pre2 catalytic helix. Solid surface (left) and backbone (right) rendering of the yeast 20S core proteasome structure with (A) WT Pup2 and (B) mutant *pup2**-10* subunits (yellow) and the closest surrounding subunit chains Pre2 (β5, purple) and Pre7 (green). In a slice of the backbone image to display Leu101 or Pro101 (right), the H1 helix (dark blue) of Pre7 (green) and the catalytic helix (cyan) of Pre2 (purple) are shifted toward the mutated Pro101 of *pup2**-10*.

Pup2 is part of the initial core assembly intermediate for the proteasomal alpha-ring and interacts strongest with the Dmp1-Dmp2 heterodimeric assembly chaperones (Yashiroda *et al.* 2008; Satoh *et al.* 2019). Importantly, the Leu101Pro point mutation in *pup2**-10* occurred at a critical residue in the H1 helix for interaction with the Dmp1-Dmp2 heterodimer in the yeast proteasome (Figure 2B, Yashiroda *et al.* 2008). To determine if the missense mutation affects protein configuration, we simulated by molecular dynamics (MD) the Leu101Pro mutation on the structure of Pup2 and the 20S core proteasome (PDB entry 1RYP, Groll *et al.* 1997). In both WT and *pup2**-10*, the residue Leu101 or Pro101 is situated at the interface between the α- and β-rings of the core particle, with the closest residues belonging to the subunits Pre7 (green) and the catalytic Pre2 (purple) (Figure 3). Compared with the wild-type structure, the mutant shows rearrangements surrounding the H1 helix in the *pup2**-10* subunit together with a movement of the H1 helix (dark blue) in Pre7 toward the Pro101 residue and a large upward shift of the catalytic helix (cyan) in Pre2 (Figure 3). These changes around the active site of neighboring Pre2 should affect catalytic function and explain the QC deficiencies in the *pup2**-10* mutant.

### Doa4 acts in CytoQC and ERAD

**Figure 4:**
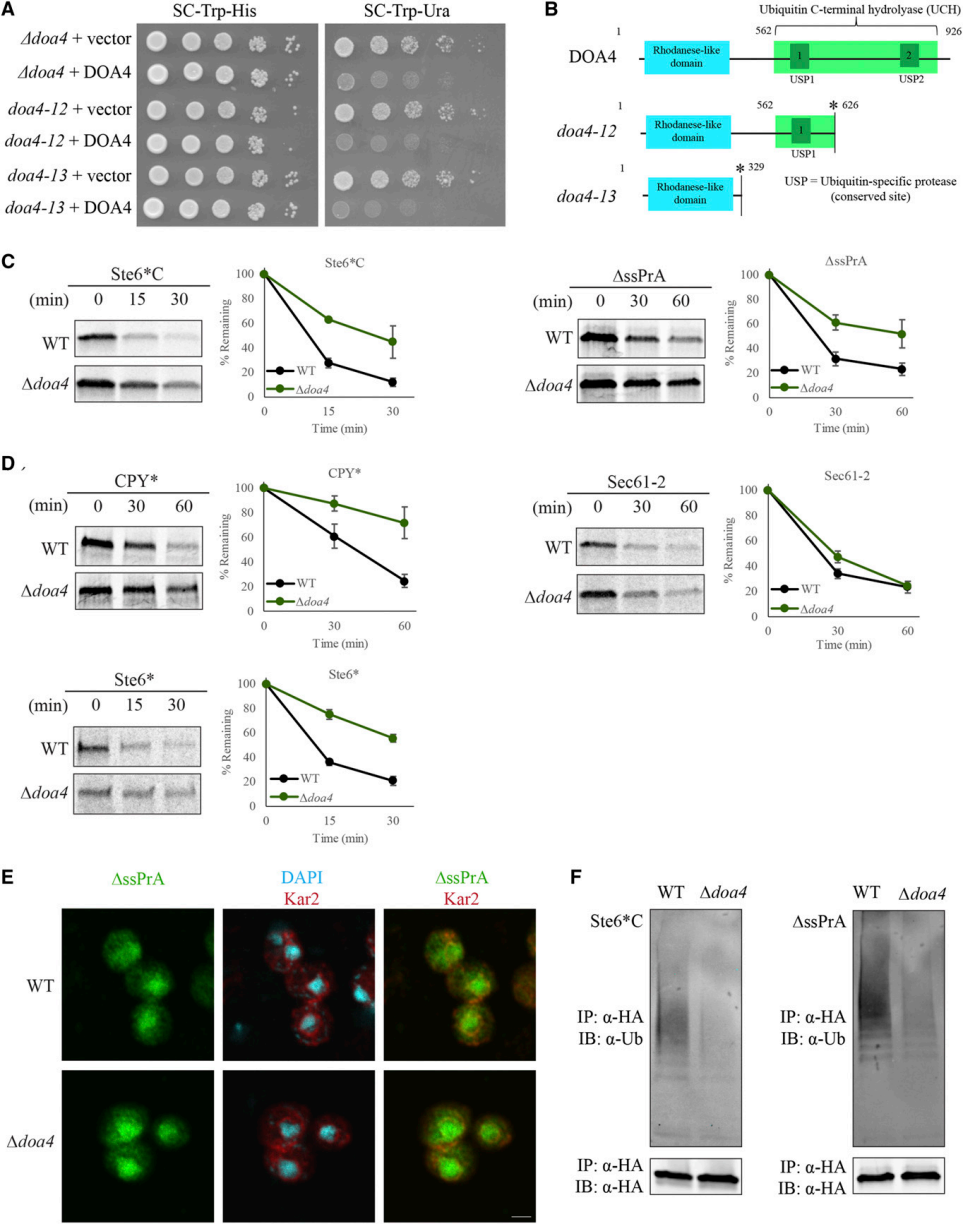
Doa4 plays a role in the degradation of CytoQC and ERAD substrates. (A) Exogenous expression of wild-type Doa4 (DOA4) in the *doa4* mutants rescues the CytoQC-defective phenotype. Equal numbers of each strain were spotted as described in Figure 2A. Vector: empty vector. (B) Nonsense mutations in two alleles of DOA4 obtained in the spontaneous mutant selection. Truncation occurs at the site labeled with the asterisk (*). (C-D) Degradation kinetics of CytoQC and ERAD substrates were determined by pulse chase assays as described in Figure 2C and D. (E) CytoQC substrate ∆ssPrA was localized predominantly in the nucleus in both WT and *∆**doa4*. Substrates were detected as described in Figure 2E. Scale bar: 2µm. (F) Reduced levels of polyubiquitinated substrates were observed in *∆**doa4*. Misfolded CytoQC substrates expressed in WT and *∆**doa4* were processed and analyzed as described in Figure 2F.

From the spontaneous mutation selection, we also obtained two mutant truncations of DOA4 encoding a deubiquitinase (Figure 4A and B) (Papa and Hochstrasser 1993). As both alleles are deletions in the catalytic domain and should not display hydrolyase activity (Figure 4B), we assayed for PQC defects with the deletion *doa4* strain (*∆**doa4*). The degradation kinetics of model substrates in *∆**doa4* were comparable to both *doa4* truncation mutants (Figure 4C and S6). Stabilization of both CytoQC substrates was observed (Figure 4C), together with ERAD substrates CPY* and Ste6* (Figure 4D), suggesting that Doa4 is involved in both CytoQC and ERAD. Surprisingly, no degradation delay of ERAD substrate Sec61-2 was observed.

To characterize the role of Doa4 in the CytoQC pathway, we localized ∆ssPrA and observed it in the nucleus of both WT and *∆**doa4* (Figure 4E). This observation suggests that nuclear import of substrates is not impeded in the absence of Doa4. In contrast to *pup2**-10*, when we assayed the ubiquitination status of misfolded Ste6*C and ∆ssPrA, we found a reduced level of polyubiquitination in *∆**doa4* compared to WT (Figure 4F and S7). Together, these data suggest that the defect in *∆**doa4* likely affects substrate polyubiquitination downstream of nuclear import.

### Degradation delays in *∆*doa4 are not solely due to reduced ubiquitin levels

**Figure 5:**
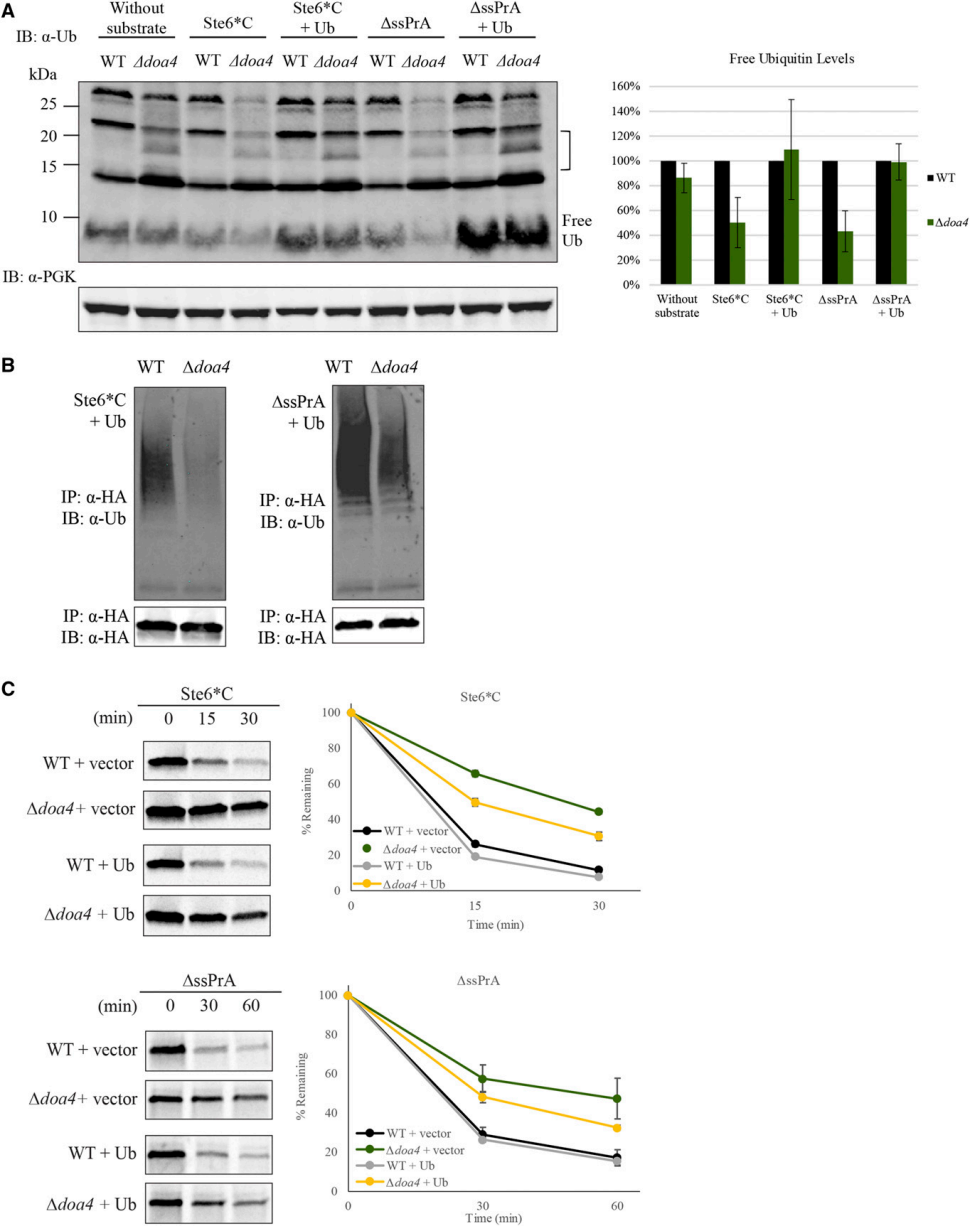
Reduced free ubiquitin levels cannot fully account for degradation delays in *∆**doa4*. (A) Immunoblot of free ubiquitin levels in WT and *∆**doa4* deletion strains without any substrate, expressing CytoQC substrates Ste6*C or ∆ssPrA, and expressing either substrate in the presence of ubiquitin overexpression (Ub). Free ubiquitin was probed with the mouse anti-ubiquitin antibody and PGK was probed as loading control. Unique ubiquitin conjugates characteristic of *∆**doa4* are indicated by the black bracket. Graph shows average relative levels of free ubiquitin in *∆**doa4* compared to the corresponding WT. Error bars indicate standard deviation of the mean of three independent experiments. (B) Partial restoration of polyubiquitinated substrate levels in *∆**doa4* with the overexpression of ubiquitin (Ub). Misfolded CytoQC substrates expressed in WT and *∆**doa4* were processed and analyzed as described in Figure 2F. (C) Degradation delay of Ste6*C and ∆ssPrA was only partially suppressed with ubiquitin overexpression. Strains were pulsed with 35S-Met/Cys as described in Figure 2C. Vector: empty vector.

As the absence of Doa4 is reported to reduce free ubiquitin levels in the cell (Swaminathan *et al.* 1999), it is plausible these levels were limiting for substrate polyubiquitination in *∆**doa4*. To test this possibility, we examined the levels of free ubiquitin in *∆**doa4* compared to WT. Consistent with previous studies (Papa and Hochstrasser 1993; Swaminathan *et al.* 1999), the unique ubiquitin conjugates of *∆**doa4* were clearly observed and the free ubiquitin level was only slightly reduced in *∆**doa4* when no exogenous misfolded substrate was expressed (Figure 5A, bracket). Nevertheless, when misfolded substrates were expressed, the free ubiquitin levels in *∆**doa4* were greatly reduced compared to WT (Figure 5A).

To assess if the reduction in free ubiquitin leads to substrate stabilization, we restored the free ubiquitin in *∆**doa4* to WT levels (Figure 5A). After overexpression of exogenous ubiquitin under a strong constitutive promoter, the free ubiquitin levels in *∆**doa4* rose to WT levels. However, the extent of polyubiquitinated CytoQC substrates increased but remained lower than WT levels (Figure 5B and S7). Likewise, only a partial rescue of the degradation delay of CytoQC substrates in *∆**doa4* was observed (Figure 5C). These data suggest that while the level of free ubiquitin does affect CytoQC, the partial rescue implicates other factors.

## Discussion

**Figure 6:**
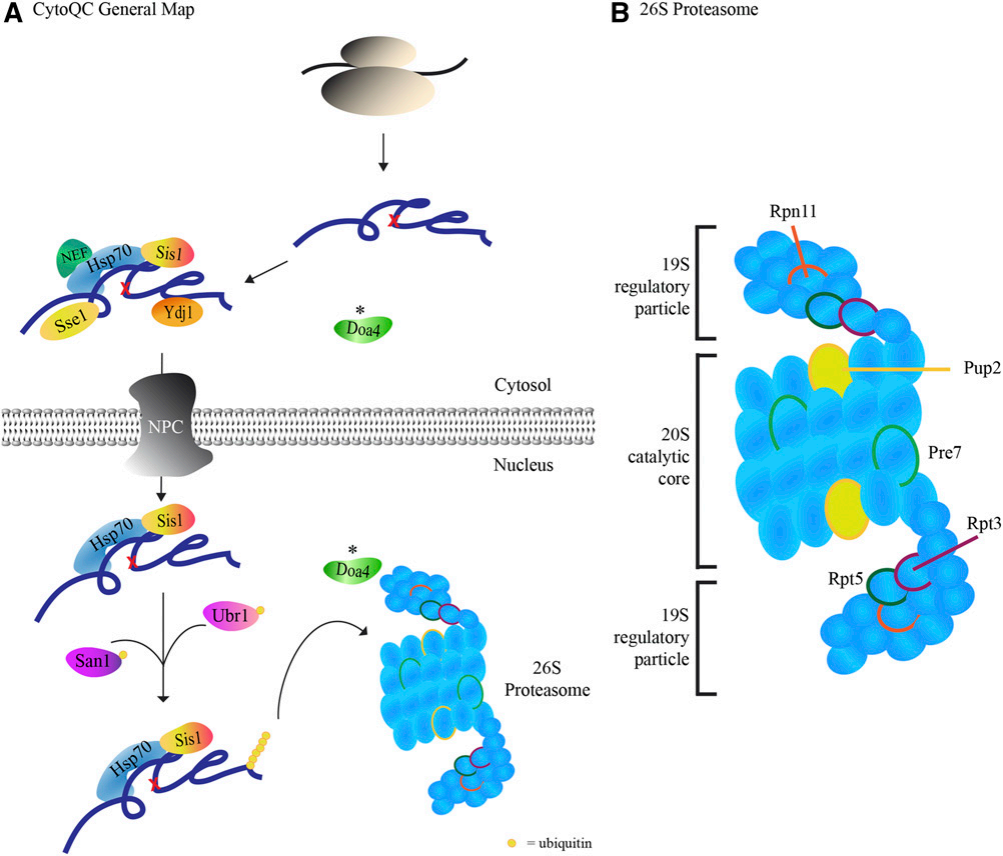
Map of the CytoQC pathway including new components from the spontaneous mutant selection. (A) General map of CytoQC pathway with new component Doa4 indicated by the asterisk (*) for misfolded CytoQC substrates which are imported into the nucleus for degradation. (B) New proteasomal-related CytoQC components and their positions in the 26S proteasome. Positions of proteasome subunits are based on Gomes (2013). Components are not drawn to scale. NPC: nuclear pore complex.

In this study, we introduced the CytoQC substrate, Ste6*C-HA-Ura3, in a broad-range genetic approach to study the CytoQC pathway. The six new CytoQC components obtained and Ubr1 are summarized in a map of the pathway in Figure 6. The two genes, PUP2 and DOA4, we validated showed that Pup2 is a proteasomal subunit that predominantly affects the degradation of CytoQC substrates while Doa4 affects the degradation of misfolded substrates in CytoQC and ERAD.

### Broad-range genetic selection uncovers new CytoQC mutants

In comparison to previous deletion library screens, our selection has several unique aspects. First, because the rate of mutations is low (Ng 2005), spontaneous mutation is not a common approach for identifying PQC genes but has shown the capability of complementing other mutagenesis approaches to uncover new components of CytoQC in this study. Furthermore, this spontaneous mutation approach minimizes the possibility of obtaining secondary mutations in the CytoQC pathway and has facilitated the identification of mutant genes. Second, we screened the genome and not a library of deletion strains as starting material for the selection. This allowed us to obtain dominant and recessive mutations across the entire genome of essential and non-essential genes. Lastly, the reporter Ste6*C-HA-Ura3 is Ubr1-dependent and the degradation of the misfolded portion, Ste6*C, involves import into the nucleus (Figure 1B, Prasad *et al.* 2018). Thus, using Ste6*C-HA-Ura3 in the selection allows us to obtain mutants in additional components that favor the nuclear import-based mechanism of CytoQC as well as upstream and downstream processes of the pathway. There is potential to identify more CytoQC genes because our selection has not reached saturation and mutants of other known genes besides UBR1 have not been isolated. In addition, we have confirmed by metabolic labeling another seven CytoQC-defective mutants but have not identified them by DNA sequencing.

### Selectivity of Pup2 for CytoQC substrates

Our preliminary analysis of model misfolded substrates in the *pup2**-10* strain clearly showed a greater stabilization of CytoQC substrates and less so for ERAD substrates compared to other mutants (Figure 2C, 2D and S3), suggesting that Pup2 is predominantly involved in the CytoQC pathway. This data supports the view that misfolded substrates could be processed differently in PQC pathways, even at the later stage of degradation. A simple explanation is that CytoQC substrates are imported into the nucleus for polyubiquitination and proteasomal degradation while ERAD substrates require retrotranslocation from the ER to the cytosol prior to degradation presumably in the cytoplasm (Ye *et al.* 2001; Hampton and Sommer 2012; Park *et al.* 2013; Prasad *et al.* 2010, 2018). In this case, a difference in overall ubiquitination or structure of the misfolded substrate and its associated components may affect how each substrate engages with the proteasome for degradation (Amm *et al.* 2014; Shiber and Ravid 2014). Based on the molecular dynamics simulations, we propose that the predicted changes in orientation of *pup2**-10* and the catalytic domain of neighboring catalytic β-subunit Pre2 are responsible for the functional deficiencies in the 20S proteasome, as portrayed by the assays of *pup2**-10*. Because Pup2 is located within the core proteasome, *pup2**-10* might affect the substrate selectivity at the translocation channel for substrates from the 19S regulatory particle to the 20S core (Finley 2009; Park *et al.* 2011; Olszewski *et al.* 2019), though further studies are required to confirm the structural effect of Leu101Pro and additional PQC substrates could be tested to verify the observed selectivity. It should be noted that the role and function of proteasomal subunits in PQC is not well elucidated and the *pup2**-10* allele could thus be a useful tool for in-depth studies of the proteasome in PQC.

### Doa4’s role in PQC affects substrate polyubiquitination

Doa4 is a deubiquitinase in ubiquitin homeostasis that is implicated in both proteasomal degradation and endocytosis (Papa and Hochstrasser 1993; Amerik *et al.* 2000, 2006). Consistent with previous studies (Papa and Hochstrasser 1993; Swaminathan *et al.* 1999), we also reported a delay in degradation of misfolded substrates in both CytoQC and ERAD (Figure 4C and D), accompanied by pronounced reductions in free ubiquitin levels in *∆**doa4* expressing misfolded substrates (Figure 5A). Given that deubiquitinases trim ubiquitin chains and potentially delay degradation of substrates (Zhang *et al.* 2013), we expected to observe an increase and not a decrease in polyubiquitinated substrates in the absence of Doa4. If the reduced free ubiquitin levels are solely responsible, restoration of ubiquitin to WT levels should have fully rescued the polyubiquitination levels and degradation delays of substrates, as reported for *∆**ubp6*, another deubiquitinase involved in ubiquitin homeostasis (Wu *et al.* manuscript in preparation). Because Doa4 is also involved in ubiquitin homeostasis, we speculated that restoring free ubiquitin to wild-type levels would restore the WT-extent of polyubiquitination and degradation. On the contrary, we showed that, other than the degradation kinetics, substrate polyubiquitination was also only partially rescued, indicating that other factors in addition to free ubiquitin impede CytoQC in *∆**doa4*.

In summary, our selection complements previous studies to uncover new CytoQC components and mutant alleles which demonstrate the potential of this approach to increase our knowledge of the CytoQC pathway. Since our current data set includes other uncharacterized mutant alleles of the proteasome (Table 1), a further in-depth study of each allele would be useful in understanding the role of these subunits in not only CytoQC, but also in general proteasome degradation. Consequently, as we saturate the selection to identify more components and make use of model substrates as reporters for other pathways, this approach will further uncover the detailed mechanisms in CytoQC.
